# The Effect of a Serious Health Game on Children’s Eating Behavior: Cluster-Randomized Controlled Trial

**DOI:** 10.2196/23050

**Published:** 2021-09-02

**Authors:** Frans Folkvord, Gosse Haga, Alexandra Theben

**Affiliations:** 1 Tilburg School of Humanities and Digital Sciences Tilburg Netherlands; 2 Open Evidence Barcelona Spain; 3 Open Evidence Research Radboud University Nijmegen Netherlands

**Keywords:** children, eating behavior, food-cues, serious health game, health intervention

## Abstract

**Background:**

Currently, children’s dietary intake patterns do not meet prescribed dietary guidelines. Consequently, childhood obesity is one of the most serious health concerns. Therefore, innovative methods need to be developed and tested in order to effectively improve the dietary intake of children. Teaching children how to cope with the overwhelming number of unhealthy food cues could be conducted effectively by serious health games.

**Objective:**

The main aim of this study was to examine the effect of a serious health computer game on young children’s eating behavior and attitudes toward healthy and unhealthy foods.

**Methods:**

A cluster-randomized controlled trial with a between-group design was conducted (n=157; 8-12 years), wherein children played a game that promoted a healthy lifestyle or attended regular classes and did not play a game (control). The game was designed in collaboration with researchers and pilot-tested among a group of children repeatedly before conducting the experiment. After 1 week of playing, attitudes toward food snacks and actual intake (children could eat *ad libitum* from fruits or energy-dense snacks) was assessed.

**Results:**

The results showed that playing a serious health game did not have an effect on attitude toward fruits or energy-dense snacks or on the intake of fruits or less energy-dense snacks. Additional Bayesian analyses supported these findings.

**Conclusions:**

Serious health games are increasingly considered to be a potential effective intervention when it comes to behavior change. The results of the current study stress the importance of tailoring serious health games in order to be effective, because no effect was found on attitude or eating behavior.

**Trial Registration:**

ClinicalTrials.gov NCT05025995; https://tinyurl.com/mdd7wrjd

## Introduction

### Background

Developing healthy eating patterns during childhood and adolescence is essential for healthy growth and development [1]. Research shows that eating behaviors established during youth continue during adulthood and play a major role in developing long-term health and chronic disease risks [2-4]. Moreover, currently, the dietary intake patterns of children and adolescents do not meet prescribed dietary standards [5,6]. As a consequence, overweight and obesity in children have greatly increased over the last three decades and are one of the most serious health concerns [7,8].

Multiple factors influence eating behaviors and food choices among children. One major contributor is food cue exposure, which can lead to actual food intake by activating both physiologic and psychological responses [9-11]. Research has shown that food cues embedded in advertising play a contributing role in this process [9-12]. Advertising in which food cues are depicted has a direct effect on preferences by creating familiar and positive associations with the brand and food products [13]. For example, food companies aim to mobilize, enhance, and even create food preferences among consumers. Messages in advertisements for snacks and high caloric food products are formulated in a way to exploit preferences for sweetness and energy density [13].

Currently, people live in a media-saturated environment, in which advertisements and food cues are omnipresent [11]. As a result, people are constantly exposed to food cues and, in particular, food cues for highly energy-dense snacks [9,10,14,15]. Due to priming-exposure to these food cues, physiological and psychological responses are automatically activated [9,10,16], making it hard for children to inhibit their responses and resist consuming palatable food [9,17]. This effect applies especially to young children because their inhibition system is still developing [9,15].

Some studies have shown that teaching children how to cope with the overwhelming number of unhealthy food cues using serious health games might be an interesting technique [18-20]. Fun elements in games attract, capture, and maintain attention, which suggests that games have the capacity to enhance exposure to health-stimulating messages [18]. Games can teach children to solve problems, gather information, analyze risks, and provide various insights in an enjoyable and educational way [20]. Games that aim to conduct this are labeled as serious health games [21,22].

### Theory

A serious health game is a rule-based system with variable outcomes, which can be altered by the performance of the player [23]. Within these rules, the player has to overcome a physical or mental challenge to reach the goal of the game [22]. Due to this control, the player becomes emotionally connected to the outcome and consequences of their own actions and decisions, giving them the feeling of being transported in the game [21]. This state of mind is called *flow*, which can lead to arousal, physical responses, enjoyment, involvement, persuasion, and memory.

The enjoyable state that players experience during playing these games can be transferred to the implicit messages that are integrated in the gaming environment [22]. That is why games are labeled as *lean-forward media* in contrast to traditional *lean-back media*, such as radio and television [24]. Consequently, serious games are considered to be highly effective in catching and retaining the attention of children; this makes games an ideal platform for communicating implicit eating behavior associations, and thereby, improving their intake [18,22].

Given the enormous amount of daily food choices, eating behavior mainly consists of automatic choices, which are highly vulnerable to situational, contextual, and implicit factors [25-27]. Several studies confirm that embedded health messages in media have the power to alter attitudes and behaviors [28-30]. Therefore, implicitly altering the attitudes and decisions with regard to food snacks in serious health games is of interest as an intervention technique [18].

The main objective of this study was to test the effectiveness of a serious health game that was specifically developed to improve eating behavior among children. The game was designed by a professional game designer based on game learning theories for enhancing playing motivation, attention, and retention of the message by manipulating game characteristics, to increase the children’s playing experience [31]. In every step of the development, the game was pilot-tested among the target age group (6-12 years) for difficulty, understanding, user-friendliness, attitude toward the game, and clarity of instructions.

### Hypotheses

The integration of food choices in videogames can transfer negative implicit associations with unhealthy food products and eating behavior, and healthy implicit associations with healthy food products and eating behavior [22]. Based on these findings, we expected that children who played the serious health game would have more positive attitudes toward fruits than those of the children who did not play the serious game (hypothesis 1a) and would have more negative attitudes toward energy-dense snacks than those of the children who did not play the serious game (hypothesis 1b). We also expected that children who played the serious health game would eat more fruit (hypothesis 2a), less energy-dense snacks (hypothesis 2b), and more in general (hypothesis 2c) than the children who did not play the serious health game, because of the effects of the food cues.

## Methods

### Experimental Design

We used a between-group design with 2 conditions. Children were randomly assigned to 1 of the 2 conditions; children who played a serious game and children who did not play a serious game. Attitudes toward foods in the game and actual food intake were assessed as the dependent variables.

### Serious Health Game: Garfield vs Hotdog

A serious health game, called Garfield versus Hotdog, was used. This game was designed by a professional game designer to increase the knowledge and awareness of children aged between 6 to 12 years about healthy food products and healthy eating habits. The game was designed in collaboration with scientific researchers based on a behavior change technique theoretical framework [32]. In addition, the game was pilot-tested among a group of children within the age of 6 and 12 years, repeatedly, in accordance with cocreation methodology. In the game, Garfield the Cat must make several cities healthy again. To make a city healthy again, the player has to play 7 different mini-games; every game has its own implicit message embedded. In general, based on the behavior change techniques taxonomy [32], most games contained mixed techniques to provide information about the link between behavior and health, provide direct information on consequences of an action, prompt intention formation, provide general encouragement through compliments and rewards, set graded tasks, provide instructions, model and demonstrate the behavior in the game, use prompt specific goal setting, prompt review of behavioral goals through the feedback if a participant is playing the game well, and provide feedback on the performance.

The first minigame teaches children how to recognize healthy and unhealthy food products. The player directs Garfield to collect healthy foods, which increase the score; collecting unhealthy foods will decrease the score. A specific score is needed to play the next level. The goal of the second minigame is to enhance self-control. The player is taught that it is better to prepare one’s own meal than it is to eat at restaurants—Garfield can only finish the level when a specific score is reached, which can be achieved if Garfield prepares his own meals. If Garfield consumes his meal at restaurants, it is more difficult to complete the level. The goal of the third minigame is to teach children that a regular and balanced diet is important, by providing points if the participant directs Garfield to do this. The fourth minigame educates children that exercising and playing outside is healthy and fun. The fifth minigame introduces the idea that a regular diet helps Garfield stay healthy, by modifying his weight and body according the choices of the participant. When the participant directs Garfield to eat regular diets, he will stay healthy and fit and fast, otherwise he will become heavier and slower. The sixth minigame aims to teach children how to recognize and protect themselves against marketing techniques used to sell unhealthy foods, by providing specific feedback on decisions children make after being exposed to advertising while playing the game and making food decisions for Garfield. The seventh minigame encourages children to drink more water on a daily basis instead of sugary sodas; Garfield’s weight increases and he becomes slower when the participant directs Garfield to drink sugary sodas. Every minigame teaches children a different healthy eating behavior, that is transferred and internalized during game play.

### Procedure

The committee for ethical concerns of the Behavioural Science Institute at the Radboud University, the Netherlands, approved this study (ECG/CW-MB/13.03). We obtained written consent from the administrators of 2 schools, chosen based on convenience sampling, and we sent the parents of children who attended these schools letters with detailed information about the study. We instructed them to inform us if they did not want their child to participate in the experiment or if their child was allergic to one of the test foods. Children who were allergic to the test food did not participate in the experiment. In total, approximately 91% of the children participated. We emphasized to the parents and the children beforehand that all data would remain confidential and children could cease participation at any time.

A between-group design was conducted with 2 conditions (control vs intervention group). Classes were randomly assigned by flipping a coin. In the control group, the experimenter explained to the class that the children would be participating in an experiment, without mentioning serious health games. After this explanation, children were tested individually in order to overcome peer influences. In the experimental condition, the experimenter presented the game to the class and explained how to download it at home and how to play it on a tablet. The children were asked to download the game and play it at home for a week. Children who were not allowed to participate in the study received a different task from the teacher to conduct outside the classroom.

After 1 week, children from the intervention group were tested. Children were tested individually at school during their regular school hours. The experimental setting which was a quiet and separate classroom. This room contained a table with a computer, 4 bowls with 4 different food products, and a line puzzle. When the child was seated, the researcher explained that the child should make the line puzzle and was free to eat *ad libitum*. The 4 bowls contained pieces of bananas, pieces of mandarins, jelly candy (cola bottles), and milk chocolate candy shells.

**Figure 1:**
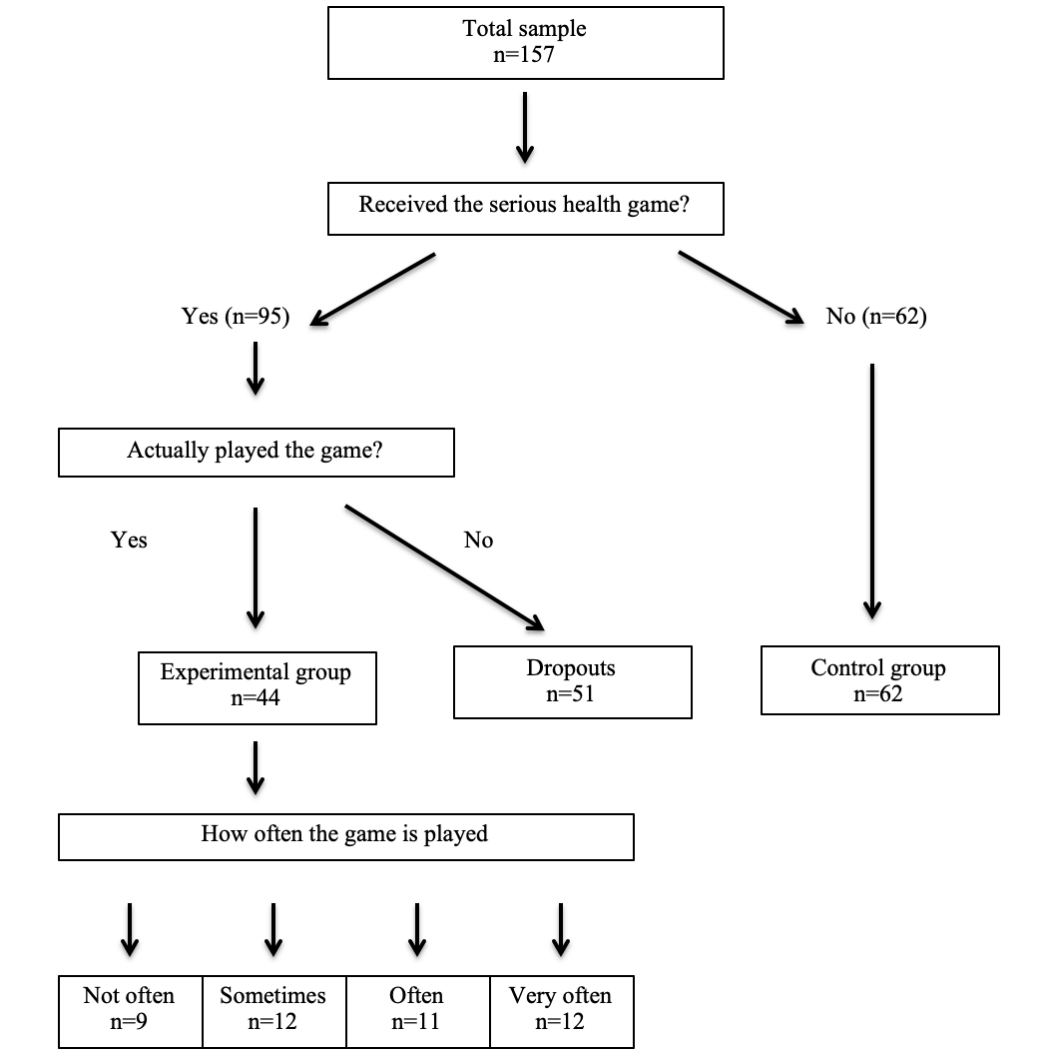
Study flow diagram.

The researcher explained that the goal was to solve the puzzle. After the participant started solving the puzzle, the researcher left the room for approximately 5 minutes. This was done to allow the child to make food choices autonomously. After 5 minutes the experimenter returned to the room. The participant’s weight and height were measured to determine BMI, and together, the researcher and child filled out a questionnaire that assessed sex, age, school year, and the attitude toward the 4 food products on the table. Additionally, the children in the experimental group were asked to what extent they liked the game, how frequently they played the game, and the perceived level of difficulty of the game. Children who received the link to download the game but did not actually play the game were considered to have dropped outs (n=51) and their data were not included in analyses; therefore, the experimental group consisted only of children who had actually played the game (Figure 1).

After filling out the questionnaires, the child returned to their classroom and the next child was tested. After each child, the 4 bowls were weighed and refilled so the next participant did not notice how much the previous participant had eaten.

### Measures

#### Attitude Toward Food Products

To measure attitude toward the fruit and energy-dense snacks in the experiment, we used a questionnaire, which has been used in previous studies [33,34]. For each food product, we asked 6 questions to measure the attitude toward food products. The 4 possible answers varied from “I totally do not like this product” to “I like this product very much.” Together, these 6 questions are reliable for each food product: banana (Cronbach α=.822), mandarin (Cronbach α=.875), candy bottles (Cronbach α=.869), and chocolate candy shells (Cronbach α=.869).

#### Food and Caloric Intake

To measure food intake, we measured the individual weight of each bowl of food. Children could eat the food products *ad libitum* during the experiment. We used a professional balance scale (precision 0.1 g). Before each child entered the room, we measured the amount of food in each bowl and weighed the bowl again after the child had completed the experiment. Therefore, we were able to calculate food intake using the nutritional values of each product [34].

#### Individual Characteristics

Age, gender, and grade were assessed using a questionnaire. We used a measuring tape to measure height (to the nearest 0.5 cm) and a professional balance scale was used to estimate weight (to the nearest 0.1 kg; children did not wear a jacket or shoes during weighing) in order to calculate BMI. We categorized children’s weights as underweight, normal weight, overweight, or obese using international cut-off scores [35]. Children with underweight or normal weight were allocated to the low BMI group and overweight and obese children were allocated to the high BMI group.

#### Perceived Difficulty and Attitude Toward the Game

Participants attitudes toward the game were assessed using 7 questions about gameplay (difficulty) and attitude toward the game (like, fun, nice, boring, stupid, annoying), with answer options that ranged from “Not at all” (1) to “Very much” (4).

### Statistical Analysis

Before testing the hypotheses, we used analysis of variance to verify whether sex, BMI, and age differed between groups (no significant differences were found—sex: *P=*.73; BMI: *P=*.29; age: *P=*.29; Table 1), and Pearson correlations between the variables were calculated. The attitude toward food products and intake of specific foods were positively correlated (banana: *r*=0.17, *P=*.02; mandarin: *r*=0.24, *P=*.001; jelly candy: *r*=0.44, *P<*.001; chocolate: *r*=0.40, *P=*.008). Attitude toward unhealthy food products were negatively correlated with age (jelly candy: *r*=−0.21, *P=*.02; chocolate: *r*=−0.20, *P=*.001) and BMI (jelly candy: *r*=−0.14, *P=*.02; chocolate: *r*=−0.22, *P=*.01). For this reason, we included age and BMI as covariates in our analyses. In addition, we computed residual scores and tested them for Mahalanobis distance, Cook distance, and leverage scores; we found no indication of outliers.

**Table 1:** Group characteristics (n=106).

Characteristic			No game (n=62)		Game (n=44)		*P* value
**Sex, n (%)**							.73
	Boy	32 (51)		22 (50)			
	Girl	30 (49)		22 (50)			
BMI^a^			18.0 (2.6)		17.5 (3.1)		*.*29
Age (years), mean (SD)			10.1 (1.2)		10.5 (1.5)		*.*29
**Attitude (rating), mean (SD)**							
	Banana	18.3 (3.7)		19.0 (3.5)		*.*17	
	Mandarin	18.4 (3.8)		17.6 (5.5)		*.*63	
	Jelly candy	20.6 (3.5)		20.8 (3.5)		*.*42	
	Chocolate	20.5 (3.9)		20.5 (3.7)		*.*91	
**Intake (grams), mean (SD)**							
	Banana	6.7 (15.9)		8.6 (19.2)		.39	
	Mandarin	2.1 (7.4)		2.0 (3.4)		*.*99	
	Jelly candy	41.5 (48.8)		41.9 (55.0)		*.*40	
	Chocolate	19.3 (28.1)		17.3 (37.7)		*.*87	

^a^BMI: body mass index.

Multivariate analysis of covariance (MANCOVA) was used to test the effect of the game on attitudes toward the fruits and energy-dense snacks, with BMI and age as covariates, and to test the effect of the game on the intake of fruits and energy-dense snacks, with BMI and age as covariates. Univariate analysis of covariance (ANCOVA) was conducted to examine the effect of the health game on general intake, controlling for BMI and age. Moreover, to further test for the (non)existence of the main effects of the experimental condition, multiple Bayesian ANCOVAs were performed with JASP software (version 0.7, Jasp project). Evidence for each model in these analyses is evaluated against the null model, which is represented by the BF_10_ value. BF_10_>3 is interpreted as substantial support for the alternative hypothesis, and BF_10_<0.33 is substantial support for the null hypothesis. BF_10_=0.33-3 suggest that the data are insensitive [36]. The data that support the findings of this study are available from the corresponding author upon reasonable request.

## Results

### General

The total sample consisted of 172 children (grades 5, 6, 7, and 8; age: mean 10.2 years, SD 1.4) from 2 Dutch primary schools. From the total number of children, 15 were excluded because their parents did not give consent for them to participate in the study. In addition, 41 children did not play the game and were excluded from the analyses. Therefore, we used a sample that consisted of 106 children (girls: 49/106, 46.4%). Of the children in the sample, 6.3% (7/106) were underweight, 74.8% (79/106) were normal weight, 14.4% (15/106) were overweight, and 4.5% (5/106) were obese. In total, 18.9% of the children (20/106) were overweight or obese, which is higher than the current national percentage of overweight and obese children in the Netherlands (12.2%). This is probably due to the fact that primary schools that were willing to participate have a relatively higher percentage of children with overweight.

### Hypothesis 1

Based on MANCOVA with BMI and age as covariates, no differences in attitude toward healthy foods (*P=*.44) or unhealthy foods (*P=*.60) were found. Age was significantly related to attitude toward jelly candy (*F*_1,105_=3.971, *P*=.049) and milk chocolate candy shells (*F*_1,105_=7.098, *P*=.009) but not attitudes toward bananas (*P=*.16) and mandarins (*P=*.91), while BMI showed no significant relationship with attitudes toward food products (healthy foods, *P=*.15; unhealthy foods, *P=*.18).

Bayesian ANCOVA findings were consistent with these results and supported evidence against the effect of the serious health game on attitude toward the fruits (BF_10_=0.208) and energy-dense products (BF_10_=0.210). These results refute hypothesis 1a and hypothesis 1b. No moderation effects between condition and age on attitudes toward the food products were found, and the frequency of playing time did not influence effect of the game (ie, the interaction was not significant *P*=*.*528).

### Hypothesis 2

MANCOVA, with BMI and age as covariates, revealed no differences in actual consumption (*P=*.38). Age seemed to be a significant covariate (*F*_1,105_=5.062, *P*=.03) of intake of jelly candy, while BMI was not significantly related to intake for any food products (bananas, *P=*.91, mandarin, *P=*.76, jelly candy, *P=*.52, milk chocolate candy shells, *P=*.82). Older children ate more jelly candies than younger children.

ANCOVA, controlling for age and BMI, showed no significant effect of the game on general intake (*P=*.38). Bayesian ANCOVA was consistent with these results and supported evidence against the effect of the serious health game on intake of fruits (BF_10_=0.210) and energy-dense products (BF_10_=0.209), and on the general intake (BF_10_=0.209). These results refute hypothesis 2a, hypothesis 2b and hypothesis 2c. No moderation effects between condition and age on actual intake were found, and the frequency of playing time did not influence effect of the game (ie, the interaction was not significant).

## Discussion

Although previous studies [18-20] have shown that serious health games can be a potential effective intervention technique for teaching children how to cope with the overwhelming number of unhealthy food cues, it has not yet been determined to what extent healthy food cues embedded in a serious health game stimulate actual healthy food intake for children. In a meta-analysis, DeSmet et al [31] found that health games have a small effect on healthy lifestyles. To the best of our knowledge, our study is the first to determine the effectiveness of a serious health game on the actual food intake of children, using a professionally designed game that aimed to improve children’s dietary intake. We expected to find an effect of playing a serious health game on the attitude toward fruits and energy-dense snacks (hypothesis 1). We also expected to find a positive effect of playing a serious health game on the actual intake of fruits and a negative effect on energy-dense snacks (hypothesis 2).

The results showed that the children who played the serious health game did not have a more positive attitude toward fruits and did not have a more negative attitude toward energy-dense snacks than children who had not played the game. Hypothesis 1a and hypothesis 1b were rejected. To examine the effect of the serious health game on the actual food intake we measured food intake of children who played the game and children who did not play the game. We did not find any differences in actual food intake between these 2 groups. This finding was applicable to all 4 food products, and intake in general. Children who played the game did not eat more healthy foods or less unhealthy foods than children who did not play the game. Hypothesis 2a, hypothesis 2b, and hypothesis 2c were rejected.

Serious health games have the capacity to enhance exposure to health-stimulating messages [18-20], but an unresolved issue is the extent to which this exposure alters real attitudes and, eventually, eating behaviors. Serious health games are designed to attract the players’ attention and simultaneously impart implicit attitudes about health behavior [37]. Eating behavior mainly consist of automatic choices which are highly vulnerable to situational, contextual and implicit factors [26]. Although the serious health game consisted of multiple implicit and explicit health messages that were directly associated with the food products that we used during the experiment, it could not stimulate healthier food choices in the children.

Games have the power to transport players into the virtual world in which they are playing at that moment. This so-called telepresence or flow, is a state of mind that gives players the feeling of getting lost in the story of the game [38]. The enjoyable state which players experience during playing games transfers to the implicit messages that are integrated in the gaming environment, which could lead to positive evaluations of these messages [22]. Therefore telepresence can lead to better product knowledge, brand attitudes, buying intentions and less counter arguing [39,40]. To reach telepresence or flow, a game has to be challenging enough and meet its player’s abilities and capacities [41-43]. This works in both ways. If a game is too easy, it is not challenging enough to get lost in the game; therefore, the player will not reach the state of telepresence. When a game is too difficult, players will quit playing, preventing them from reaching the favorable state of mind. Thus, if researchers want to intervene in children’s eating habits using games, they have to ensure players reach this state of mind. Only then can players’ attitudes and behaviors be altered. While individual tailored games could be a theoretical solution to overcome this problem [31,44], actual practical implications make this extremely difficult because serious health games need to be developed for a large group of players to be cost-effective.

Serious health games communicate healthy messages to the player with the goal to alter the players’ attitudes and behaviors. Some serious health games are not challenging enough to give players the feeling of getting lost in the story of the game which is essential in transferring implicit and explicit beliefs to the player [18]. They therefore do not have the power to alter the players’ attitudes and behaviors and need to be better adapted to children’s capabilities and skills.

The first strength of this study is that, until now, no studies have focused specifically on serious games and actual eating behavior of children. Although games are popular among children and have potential for being an effective intervention technique, minimal scientific research has been conducted on this type of media in combination with eating behaviors. Second, this study tested the effects of a serious health game in their own private environment and time, thereby increasing the ecological validity of the study. Children received a hyperlink to download the game on their tablet at home so they could play it at free will without the effects of an experimenter during playtime. We did this to increase ecological validity of the study.

One limitation of this study is that many of the children who were allocated to the experimental condition did not play the game (over half of the children). In addition, children who played the game did not play the game very often. Nonetheless, this can be considered an important finding; the adoption and frequency of playing the serious health game is an important element of its effectiveness. Second, children could only play the game for 1 week. A longer duration might have led to different results on the effectiveness of serious games on eating behavior, although the results showed that only a small group of the children played the game often or very often. Normally the game, which we provided for free during this study, has to be bought online in an app store. Given that, when children or parents have to pay, the adoption level will be even lower, it is important to investigate the adoption of serious games that aim to improve eating behavior or children. A third limitation is that we only assessed the intake of 4 different foods, whereas normally children can select many different foods as a snack. Nonetheless, these are popular food fruits [45] and candies [46] in the Netherlands among the target group. Future research might focus on whether devaluation of the unhealthy foods and more positive evaluation of healthier foods through serious health games helps children to overcome the undesired effects of food cue exposure.

In conclusion, this study did not find an effect of serious health games aiming to improve eating behavior among children. The findings further provide evidence that the adoption, the frequency of play time, and the play experience of the serious health game are important. It is important to obtain greater insight into the link between reactivity to food cues and executive control abilities in children. Unhealthy food cues that trigger eating behavior are omnipresent, and children are susceptible to these food cues [9]. In order to improve the dietary intake of children, effective intervention techniques are important.

## Abbreviations

ANCOVA: analysis of covariance
BMI: body mass index
MANCOVA: multivariate analysis of covariance

## References

[1] Story MT, Neumark-Stzainer DR, Sherwood NE, Holt K, Sofka D, Trowbridge FL, Barlow SE (2002). Management of child and adolescent obesity: attitudes, barriers, skills, and training needs among health care professionals. Pediatrics.

[2] Kaikkonen JE, Mikkilä V, Magnussen CG, Juonala M, Viikari JSA, Raitakari OT (2013). Does childhood nutrition influence adult cardiovascular disease risk?--insights from the Young Finns Study. Ann Med.

[3] Patton GC, Coffey C, Carlin JB, Sawyer SM, Williams J, Olsson CA, Wake M (2011). Overweight and obesity between adolescence and young adulthood: a 10-year prospective cohort study. J Adolesc Health.

[4] Guo SS, Roche AF, Chumlea WC, Gardner JD, Siervogel RM (1994). The predictive value of childhood body mass index values for overweight at age 35 y. Am J Clin Nutr.

[5] Neumark-Sztainer D, Story M, Hannan PJ, Croll J (2002). Overweight status and eating patterns among adolescents: where do youths stand in comparison with the healthy people 2010 objectives?. Am J Public Health.

[6] Nicklas TA, Elkasabany A, Srinivasan SR, Berenson G (2001). Trends in nutrient intake of 10-year-old children over two decades (1973-1994) : the Bogalusa Heart Study. Am J Epidemiol.

[7] Han JC, Lawlor DA, Kimm SYS (2010). Childhood obesity. Lancet.

[8] Karnik S, Kanekar A (2012). Childhood obesity: a global public health crisis. Int J Prev Med Jan 1.

[9] Folkvord F, Anschütz DJ, Boyland E, Kelly B, Buijzen M (2016). Food advertising and eating behavior in children. Curr Opin Behav Sci.

[10] Folkvord F (2019). The Psychology of Food Marketing and Overeating.

[11] Vukmirovic M (2015). The effects of food advertising on food-related behaviours and perceptions in adults: a review. Food Res Int.

[12] Story M, Kaphingst KM, Robinson-O'Brien R, Glanz K (2008). Creating healthy food and eating environments: policy and environmental approaches. Annu Rev Public Health.

[13] Hawkes C, Smith TG, Jewell J, Wardle J, Hammond RA, Friel S, Thow AM, Kain J (2015). Smart food policies for obesity prevention. Lancet.

[14] Harris JL, Speers SE, Schwartz MB, Brownell KD (2012). US food company branded advergames on the internet: children's exposure and effects on snack consumption. J Child Media.

[15] Boyland EJ, Nolan S, Kelly B, Tudur-Smith C, Jones A, Halford JC, Robinson E (2016). Advertising as a cue to consume: a systematic review and meta-analysis of the effects of acute exposure to unhealthy food and nonalcoholic beverage advertising on intake in children and adults. Am J Clin Nutr.

[16] Nederkoorn C, Jansen A (2002). Cue reactivity and regulation of food intake. Eat Behav.

[17] Folkvord F, Anschütz DJ, Nederkoorn C, Westerik H, Buijzen M (2014). Impulsivity, "advergames," and food intake. Pediatrics.

[18] Hube W, Sponholz H, Klinker L (1989). [Clinical-experimental study on the temperature behaviour of the mucosa of mouth related to the season of the year]. Stomatol DDR.

[19] Barthel ML (2013). President for a day: video games as youth civic education. Inf Commun Soc.

[20] Gee JP (2003). What video games have to teach us about learning and literacy. Comput Entertain.

[21] Shrum L (2012). The Psychology of Entertainment Media - Blurring the Lines Between Entertainment and Persuasion.

[22] Baranowski T, Buday R, Thompson DI, Baranowski J (2008). Playing for real: video games and stories for health-related behavior change. Am J Prev Med.

[23] Juul J (2003). The game, the player, the world: looking for a heart of gameness. https://www.jesperjuul.net/text/gameplayerworld/.

[24] Katz H (2016). The Media Handbook: A Complete Guide to Advertising Media Selection, Planning, Research, and Buying.

[25] Wansink B, Sobal J (2016). Mindless eating. Environ Behav.

[26] Cohen DA, Babey SH (2012). Contextual influences on eating behaviours: heuristic processing and dietary choices. Obes Rev.

[27] Wansink B (2004). Environmental factors that increase the food intake and consumption volume of unknowing consumers. Annu Rev Nutr.

[28] Folb K (2000). ´Don't touch that dial!´: TV as a--what!?--positive influence. SIECUS Report.

[29] Folkvord F, Anschütz DJ, Buijzen M (2020). Attentional bias for food cues in advertising among overweight and hungry children: an explorative experimental study. Food Qual Prefer.

[30] Hollands GJ, Prestwich A, Marteau TM (2011). Using aversive images to enhance healthy food choices and implicit attitudes: an experimental test of evaluative conditioning. Health Psychol.

[31] DeSmet A, Van RD, Compernolle S, Baranowski T, Thompson D, Crombez G, Poels K, Van LW, Bastiaensens S, Van CK, Vandebosch H, De BI (2014). A meta-analysis of serious digital games for healthy lifestyle promotion. Prev Med.

[32] Abraham C, Michie S (2008). A taxonomy of behavior change techniques used in interventions. Health Psychol.

[33] Pecheux C, Derbaix C (1999). Children and attitude toward the brand: a new measurement scale. J Advert Res.

[34] Folkvord F, Anschütz DJ, Buijzen M, Valkenburg PM (2013). The effect of playing advergames that promote energy-dense snacks or fruit on actual food intake among children. Am J Clin Nutr.

[35] Cole TJ, Bellizzi MC, Flegal KM, Dietz WH (2000). Establishing a standard definition for child overweight and obesity worldwide: international survey. BMJ.

[36] Dienes Z (2014). Using Bayes to get the most out of non-significant results. Front Psychol.

[37] Thompson D (2012). Designing serious video games for health behavior change: current status and future directions. J Diabetes Sci Technol.

[38] Green MC, Clark JL (2013). Transportation into narrative worlds: implications for entertainment media influences on tobacco use. Addiction.

[39] Kim T, Biocca F (1997). Telepresence via television: two dimensions of telepresence may have different connections to memory and persuasion. J Comput Mediat Commun.

[40] Cheng M, Chen J, Chu S, Chen S (2015). The use of serious games in science education: a review of selected empirical research from 2002 to 2013. J Comput Educ.

[41] Chen J (2007). Flow in games (and everything else). Commun ACM.

[42] Sears A, Jacko J (2009). Why we play: affect and the fun of games: designing emotions for games, entertainment interfaces, and interactive products. Human-Computer Interaction.

[43] Nakamura J, Csikszentmihalyi M (2014). The concept of flow. Flow and the Foundations of Positive Psychology.

[44] Boeije H, Hart H, Hox J (2009). Onderzoeksmethoden.

[45] Top-10 populairste verse fruit in Nederland. NAGF.

[46] Top 10 best verkochte snoepmerken. Top-10-lijstjes.

